# Effect of myricetin on the gene expressions of NOX3, TGF-β1, prestin, and HSP-70 and anti-oxidant activity in the cochlea of noise-exposed rats

**DOI:** 10.22038/IJBMS.2020.41007.9693

**Published:** 2020-05

**Authors:** Maryam Bahaloo, Mohammad Ebrahim Rezvani, Ehsan Farashahi Yazd, Fatemeh Zare Mehrjerdi, Mohammad Hossein Davari, Ali Roohbakhsh, Abolfazl Mollasadeghi, Haniyeh Nikkhah, Maryam Vafaei, Amir Houshang Mehrparvar

**Affiliations:** 1 Industrial Diseases Research Center, Shahid Sadoughi University of Medical Sciences, Yazd, Iran; 2 Department of Physiology, Shahid Sadoughi University of Medical Sciences, Yazd, Iran; 3 Stem Cell Biology Research Center, Yazd Reproductive Sciences Institute, Shahid Sadoughi University of Medical Sciences, Yazd, Iran; 4 Department of Occupational Medicine, Shahid Sadoughi University of Medical Sciences, Yazd, Iran; 5 Pharmaceutical Research Center, Pharmaceutical Technology Institute, Mashhad University of Medical Sciences, Mashhad, Iran

**Keywords:** Anti-oxidants, Gene expression, Myricetin, NADPH oxidase, Noise-induced hearing loss, Superoxide dismutase

## Abstract

**Objective(s):**

Noise-induced hearing loss is one of the most common occupational diseases in industrialized countries and can be affected by various environmental and genetic factors. This study was designed to examine the effect of myricetin in preventing this disorder.

**Materials and Methods:**

Twenty-one Wistar rats were randomly divided into five groups: Non-exposed, noise exposure only, noise exposure with vehicle, noise exposure with myricetin 5 mg/Kg, and noise exposure with myricetin 10 mg/kg. All animals were sacrificed after last noise exposure. The left cochlea was dissected from each rat. It was used for mRNA expression analysis (NOX3, TGF-β1, prestin, and HSP-70). Blood samples were collected to assess superoxide dismutase (SOD) activity, 1, 1 diphenyl picrylhydrazyl (DPPH), and malondialdehyde (MDA) measurements.

**Results:**

Real time-PCR assay revealed that noise decreased NOX3 and increased TGF-β1, prestin, and HSP-70 gene expressions. Administration of myricetin at the dose of 5 mg/kg, but not at 10 mg/kg, significantly reversed these changes. Noise also increased MDA levels and decreased SOD and DPPH scavenging activities. Myricetin at the doses of 5 and 10 mg/kg also reversed these changes.

**Conclusion:**

The findings of this study showed that myricetin at the dose of 5 mg/Kg was able to reverse noise-induced abnormalities in gene expression and oxidant/anti-oxidant balance. It is a possibility that myricetin via enhancement of anti-oxidant activity induced these effects.

## Introduction

Exposure to noise intensities higher than the permissible level, is among the most common factors with damaging effects on hearing (1). Ears cannot tolerate frequent or prolonged exposure to noise, and exposure to high levels of noise can damage sensory hair cells in the cochlea, inducing noise-induced hearing loss (NIHL) (2). NIHL has been reported as one of the most common occupational diseases in industrialized countries. 

Different mechanisms, such as mechanical stress, oxidative stress, and free radical production have been proposed as the cause of NIHL. Recently, in order to find a practical method to prevent NIHL, scientists have paid attention to the role of genetic susceptibilities and application of different chemicals, especially anti-oxidants, for the prevention of NIHL (3).

Malondialdehyde (MDA) is the final product of lipid peroxidation which is used for the evaluation of oxidative stress (4). Noise exposure as an inducer of oxidative stress, increases MDA levels (5). To counteract these conditions, the enzymatic defense system will be activated. One of these defending enzymes is superoxide dismutase (SOD), which protects cells from the harmful effects of superoxide anions (6). Another factor for assessment of anti-oxidant activity of natural compounds is 1,1 diphenyl picrylhydrazyl (DPPH) (7). 

Among genes that are considered to be related to NIHL, NOX3 gene is one of the most important genes. This gene is one isomere of the NADPH (nicotinamide adenine dinocoids phosphate) oxidase enzyme family which includes seven enzymes producing superoxides: NOX1, NOX2, NOX3, NOX4, NOX5, DOUX1, and DOUX2 (8). It was reported that expression of NOX3 was reduced after noise exposure. So, it has been suggested as an endogenous protective factor against reactive oxygen species (ROS) generation, as a mechanism of NIHL (9). 

Transforming growth factor β1 (TGF-β1) is a multi-functional peptide with unclear effects on the cochlea. Over-expression of TGF-β1 in the inner ear has been proposed as a factor of susceptibility to otosclerosis and NIHL (10).

Noise exposure can increase the expression of prestin mRNA in rats and guinea pigs (11), which may damage proteins and decrease the electromotility of outer hair cells (OHC) (12). Heat shock proteins (HSP) are a group of proteins that protect inner ear against acoustic trauma and ototoxic agents (13). 

Considering that one of the mechanisms of NIHL occurrence is oxidative stress and increased free radical production, the use of anti-oxidants by reducing ROS will be an effective step in the prevention of NIHL (14). Resveratrol (15), vitamins E and C, and magnesium (16), N-acetyl cysteine (17), statins (18), and ascorbic acid (19) are examples of these anti-oxidants used in previous studies.

Myricetin belongs to the polyphenols family and is found in berries, grapes, tea, fruits, and some medicinal plants (20). It is a potent anti-inflammatory and anti-oxidant agent with preventive effects on neurodegenerative and inflammatory diseases (21).

This study was designed to examine the effect of myricetin on NOX3, TGF-β1, prestin, and HSP-70 genes expression and SOD and DPPH scavenging activities in rats as factors that may prevent NIHL.

## Materials and Methods

***Animals***

The protocol used in this study was approved by the ethics committee of Shahid Sadoughi University of Medical Sciences. We tried to use the minimum number of animals and minimize their suffering during the study. Animals included 2 month-old male Wistar rats (weighing 200–250 g at study onset) obtained from animal house of Shahid Sadoughi University of Medical Sciences. Animals were housed in standard plexi-glass cages with controlled temperature (22–23 ^°^C) and humidity (60±5%), under a 12 hr light/dark cycle with food and water available. 

Twenty-one Wistar rats were randomly divided into the following groups: 

1. Control group: Non-exposed (n=3);

2. Noise group: Noise exposure only (n=6); 

3. Noise+Veh group Noise exposure with vehicle (n=4); 

4. Noise+Myrc5 group: Noise exposure with myricetin (Sigma-Aldrich GmbH) 5 mg/kg, (n=4); 

5. Noise+Myrc10 group: Noise exposure with myricetin 10 mg/kg (n=4). 

Myricetin and vehicle were administered from 3 days before exposure to noise and continued for 13 days. Myricetin was injected IP 1 hr before exposure to noise.

***Noise exposure***

Animals were exposed to a 10 KHz octave band noise with the intensity of 100 dB sound pressure level (SPL) for 1 hr each day for 10 consecutive days (22) in a plexi-glass box. Noise was generated by an audiometer (OB 929, Madsen, Denmark). Sound level was measured using a sound level meter (TES-1351 digital sound level meter, Taiwan).

***Tissue preparation***

All Animals were sacrificed after the last noise exposure. The left cochlea was dissected from each rat and used to determine the gene expression levels and assessment of oxidant biomarkers. 

***Gene expression assessment***

In order to determine the effect of different doses of myricetin on noise-exposed cochlea, mRNA expressions of NOX3, TGF-β1, prestin, and HSP-70 genes in the cochlea were assessed by semi-quantitative real-time polymerase chain reaction (PCR) (Qiagen, Germany). Total RNA was isolated using the RNX Plus Kit (Cinnaclon, Iran). cDNA was synthesized using a cDNA synthesis kit (Thermo Fisher Scientific, Lithuania). Real time PCR products were detected using SYBR Green and were normalized by β-Actin expression. The PCR cycling conditions included one cycle with 95 ^°^C for 10 min (denaturation I) then 40 cycles in the following steps: 95 ^°^C for 15 sec (denaturation II), 60 ^°^C for 30 sec (annealing), and 72 ^°^C for 30 sec (extension). Primers were designed using the Beacon designer software package (Premier Biosoft) and synthesized by Integrated DNA Technologies (BVBA, Leuven, Belgium). The primer sequences are presented in Table 1.

***Biochemical analysis***

Blood samples were collected for measuring the serum levels of MDA and DPPH and activity of SOD. The samples were centrifuged at 3500 rpm for 15 min to separate the serum, and the serum was then stored at -80 ^°^C until the time of analysis. SOD activity and blood concentration of MDA were measured with a commercial assay kit (Zell Bio, Germany) according to the manufacturer’s instructions. 

***Anti-oxidant activity assay for DPPH***

To determine the ability of myricetin to scavenge DPPH radicals, freshly prepared methanol DPPH (0.1 mM/ml) solution was added to different concentrations of myricetin. After half an hour, the absorbance ability was recorded at 517 nm. Results were expressed as percentage of inhibition (Inhibition (%) = [(A Control – A Extract) / A Control] ×100)

A blank represents the measured absorbance of the sample containing control reaction (all reagents without the test compound). A sample represents the measured absorbance of the test compound (26). 

***Statistical analysis***

The data were expressed as mean±SEM (standard error of mean) and analyzed using graphPad prism 7.0 (GraphPad, Inc., USA) statistical software. Kruskal-Wallis test was used to determine the significant differences between groups and was followed by Tukey’s test for *post-hoc *comparisons. *P*-value<0.05 was considered as statistical significance. 

## Results

**Figure 1:**
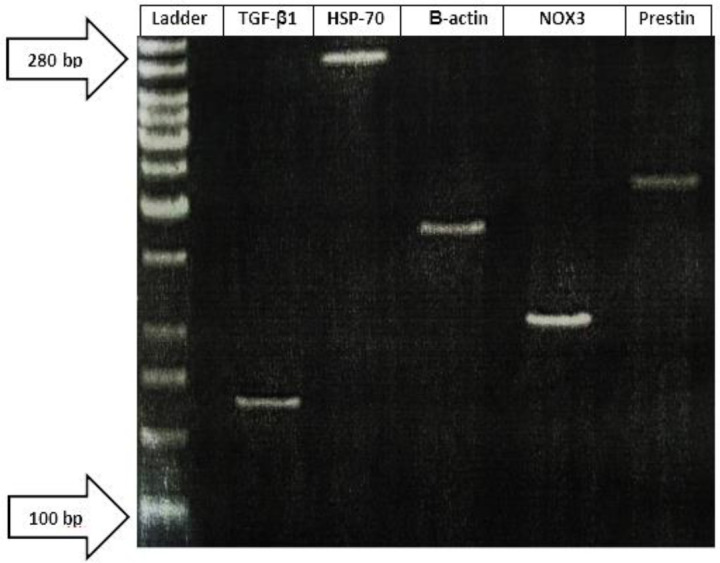
The polymerase chain reaction (PCR) reactions for non-template control were demonstrated using the agarose gel electrophoresis (10bp DNA ladder and 8% agarose gel). As shown, the individual amplifications matched the predicted sizes

Real time-PCR assay revealed that NOX3 expression was significantly decreased in noise and vehicle groups; however in Myrc 5 mg group, it was significantly increased. Expressions of TGF-β1, prestin, and HSP-70 genes were significantly increased in the noise group and was decreased in Myrc groups, but the difference was significant only for Myrc 5 mg group. Figure 1 compares the effect of myricetin on NOX3, TGFβ1, prestin, and HSP-70 gene expression in different study groups. 

**Figure 2:**
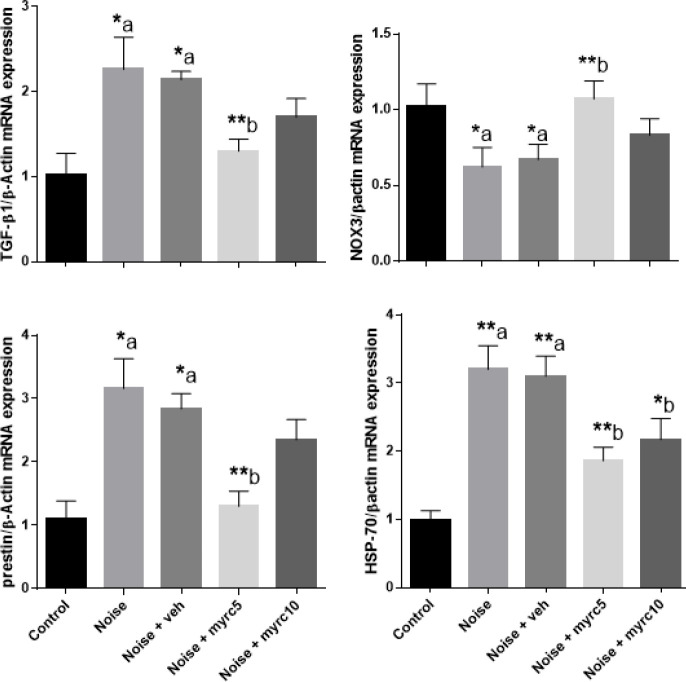
Effect of myricetin on relative gene expression of TGF-β1, NOX3, prestin, and HSP-70. Beta actin was used for housekeeping gene. One-way ANOVA were used for statistical comparisons. Data was shown as means±SEM. *P<0.05 and **P<0.01. "a" and "b" when compared to Control or Noise group respectively

Figure 2 compares the SOD activity, DDPH scavenging activity, and MDA level in different groups. 

**Figure 3:**
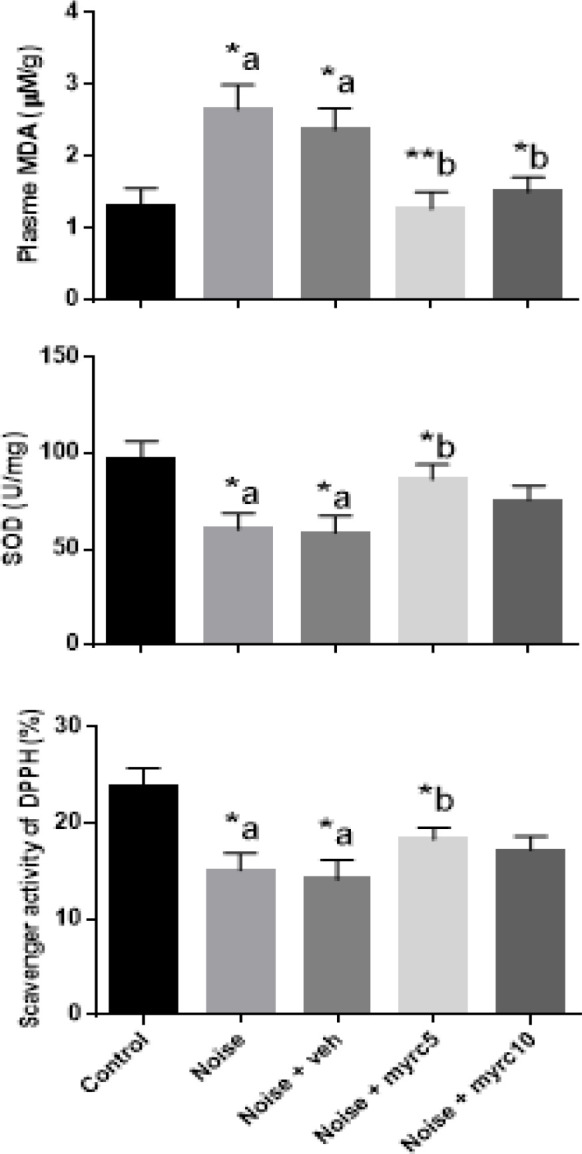
Effect of myricetin on plasma Malondialdehyde (MDA) level, superoxide dismutase (SOD) activity, and 1,1 diphenyl picrylhydrazyl (DPPH) scavenging activity in different groups. One-way ANOVA was used for statistical comparisons. Data was shown as means±SEM. *P<0.05 and **P<0.01. "a" and "b" when compared to Control or Noise group, respectively

Figure 3 compare the plasma MDA level, SOD activity, and DPPH scavenging activity in different groups.

## Discussion

NIHL is a complex disease that is affected by personal, environmental and genetic backgrounds. Workers with similar exposure to noise may show different levels of hearing damage, which proposes a genetic predisposing role (27).

Exposure to noise induces production of ROS and may lead to hearing loss (28). Present evidence suggests that anti-oxidants can prevent NIHL (29). Myricetin is an anti-oxidant and anti-inflammatory flavonoid that has ability to scavenge ROS and shows anti-oxidant activity (21). In the current study, we assessed the anti-oxidant effects of myricetin on prevention of NIHL.

Previous studies have suggested that the NADPH family can be the source of ROS production and induction of apoptosis in various cells (30, 31). NOX3 is expressed in the inner ear (32). 

Our results showed that NOX3 gene expression was reduced following exposure to noise, and administration of myricetin enhanced its expression. In agreement with the present findings, a study by Vlajkovic *et al.* evaluated the role of the NADPH oxidase family and reported that during prolonged exposure to noise, the expression levels of NOX1 and NOX2 genes increased, but NOX3 gene expression decreased. This may be a putative mechanism against oxidative stress after exposure to noise in the cochlea (9). In a study that completely evaluated the role of NOX3 in the development of NIHL, it was shown that NOX3 deficiency or absence in the inner ear increased susceptibility to NIHL (8). However, during cisplatin-induced ototoxicity, NOX3 has been reported as an inducing factor for other genes that ultimately lead to cochlear damage (31, 33). 

It has been reported that cochlear injury due to neomycin (34) and noise (35) elevated TGF-β1 activation in the cochlea, so inhibition of TFG-β1 may prevent cochlear damage due to exposure to noise or ototoxic agents. Losartan as a blocker of TFG-β1 signaling prevented axonal sprouting in the cochlea after hearing loss (36). Similarly, in the present study, myricetin decreased TGF-β1 expression. However, there are controversial reports regarding the effect of TGF-β1 inhibition on the prevention of NIHL. Murillo *et al.* showed that TGF-β1 inhibitor peptides, if used before exposure to noise, improved hearing threshold and degenerative changes due to noise exposure in a dose-dependent manner (10), but single dose of these chemicals did not prevent NIHL (35).

Prestin is a protein located in OHC lateral membrane and presents in the inner ear (11, 12). It acts as a motor protein and is necessary for OHC electromotility (37). Expression of prestin increases after exposure to noise and returns to its basal level after a few weeks, which may damage proteins and lead to cochlear damage (12). In the present study, exposure to noise increased the expression of the prestin gene, which was reversed in the myricetin-treated groups. It has been proposed as a mechanism to prevent NIHL (38). 

Environmental stresses such as hyperthermia, ischemia, and exposure to noise can induce HSP-70 (39, 40). Administration of HSP-70 may protect inner hair cells from severe ototoxic injury (41). This study showed that noise significantly increased the expression of HSP-70, but myricetin decreased its expression. This finding shows that anti-oxidant effect of myricetin decreased the oxidant stress and eventually the need for expression of HSP-70, which is a cytoprotective agent in the cochlea.

During oxidative stress, SOD activity is increased (42). SOD is an anti-oxidant enzyme in the mitochondria. It plays an important role in preventing noise-induced damage to the cochlea by ROS inhibition (43). It has been reported that lack of SOD makes young mice susceptible to NIHL (44). Exposure to noise in tile workers reduced SOD levels as well (45). In the current study, anti-oxidant effects of myricetin increased the activity of SOD in the Myrc 5 mg group. Other anti-oxidants, such as D-methionine (46), vitamin E (47), and Rehmannia Glutionosa, a traditional medicine (48), also increased the activity of erythrocyte SOD. In addition, myricetin increased the levels of anti-oxidant enzymes such as SOD and prevented the effects of 7.12 dimethyl benzanthracene (a breast cancer agent) on mammary tissue in female rats (49).

A study by Yildirim *et al.* showed that noise exposure in tile workers increased MDA levels due to oxidative stress in the cochlea (45). In a similar study, exposure to intense noise increased lipid peroxidation and MDA levels in the blood and administration of vitamin E decreased the MDA level (47). Similar to these studies, the present results showed that noise increased the blood level of MDA in rats, which was decreased in myrectin-treated animals. 

DPPH assay is a common method for assessing free radical scavenging capacity in natural compounds. In DPPH assay, a natural compound transfers electron or hydrogen atoms and changes DPPH activity (50). According to this mechanism, we assessed DPPH free radical scavenging capacity of myricetin. DPPH free radical scavenging capacity decreased in noise group that was reversed by administration of low dose of myricetin. Scavenging effects of APP3 (a mushroom with anti-oxidant effects) was positively correlated with concentration and it inhibited the generation of hydroxyl radicals (50).

This study showed that administration of 5 mg/Kg myricetin had a more favorable effect on NIHL prevention in genetic and biochemical levels than higher doses of this substance, which is probably due to systemic toxicity in higher doses. Similarly, N-acetyl cysteine also prevented NIHL at relatively low doses and resulted in systemic toxicity at higher doses (51).

**Table 1:** The primer sequences of different genes for real-time PCR analysis

Gene	Primers sequence (5 → 3)	Size (bp)	Annealing °C
Rat NOX3 (9)	F: ATCTTTATCCAGTGCCCATCC	**129**	**59**
	R: CTTCAGTAACGCCTCTGTCCA		
Rat TGFβ1 (23)	F: GTCAACTGTGGAGCAACACG	**116**	**61**
	R: CGTCAAAAGACAGCCACTCA		
Rat Prestin (24)	F: CACAGAGTCCGAGCTACACAGTC	**159**	**60**
	R: TCAGTGCGCTGCTGTACAAG		
Rat HSP-70 (25)	F: CTCGTGCGTGGGCGTGTTCC	**285**	**61**
	R: TTTCCCTTGTAGTTCACCTG		
Rat β-Actin (23)	F: CTATCGGCAATGAGCGGTTCC	**146**	**61**
	R: TGTGTTGGCATAGAGGTCTTTACG		

The relative gene expression was measured by the 2-∆∆Ct method and then normalized to the expression levels of endogenous β-Actin gene

## Conclusion

The findings of this study showed that noise exposure increased the expression of TGF-β1, prestin, and HSP-70 genes and MDA levels and decreased the expression of NOX3, SOD activity, and DPPH scavenging activity in rats, but administration of 5 mg/Kg myricetin in noise-exposed rats significantly reversed these effects. Therefore, myricetin at dose of 5 mg/Kg can probably be used as an anti-oxidant in prevention of NIHL. 

## Limitations

This study had some limitations: Due to monetary constraints, we couldn’t perform Western blot, which is a more accurate method.
